# Contextual determinants of CHILDREN’S health care and policy in Europe

**DOI:** 10.1186/s12889-019-7164-8

**Published:** 2019-06-27

**Authors:** Kinga Zdunek, Peter Schröder-Bäck, Denise Alexander, Michael Rigby, Mitch Blair

**Affiliations:** 1 Public Health Department, Faculty of Health Sciences, Medical University of Lublin, 1 Chodźki Street, 20-093 Lublin, Poland; 2 Department of International Health, Care and Public Health Research Institute (CAPHRI), Maastricht University, Maastricht, The Netherlands; 3 Department of Paediatrics, Imperial College London, London, UK

**Keywords:** Context, Health policy, Contextual determinants, Policy drivers

## Abstract

**Background:**

The main objective of this study was to explore the contextual determinants of child health policies.

**Methods:**

The Horizon 2020 Models of Child Health Appraised (MOCHA) project has one Country Agent (CA) in all 30 EU and EEA countries. A questionnaire designed by MOCHA researchers as a semi-structured survey instrument asked CAs to identify and report the predominating public and professional discussions related to child health services within the last 5 years in their country and the various factors which may have influenced these. The survey was issued to CAs following validation by an independent Expert Advisory Board. The data were collected between July and December 2016.

The data was qualitatively analysed using software Nvivo11 for data coding and categorization and constructing the scheme for identified processes or elements.

**Results:**

Contextual determinants of children’s health care and policy were grouped into four categories. 1) Socio-cultural determinants: societal activation, awareness, communication, trust, freedom, contextual change, lifestyle, tolerance and religion, and history. 2) Structural determinants which were divided into: a) external determinants related to elements indirectly correlated with health care and b) internal determinants comprising interdependent health care and policy processes. 3) International determinants such as cross-nationality of child health policy issues. 4) The specific situational determinants: events which contributed to intensification of debates which were reflected by behavioural, procedural, institutional and global factors.

**Conclusions:**

The influence of context across European countries, in the process of children’s health policy development is clearly evident from our research. A number of key categories of determinants which influence child health policy have been identified and can be used to describe this context. Child health policy is often initiated in reaction to public discontentment. The multiple voices of society resulted, amongst others, in the introduction of new procedures, action plans and guidelines; raising levels of awareness, intensifying public scrutiny, increasing access and availability of services and provoking introduction of structural changes or withdrawing unfavourable changes.

## Background

In Europe there is no doubt that health is a child’s fundamental right [1]. Improvement of child health can be seen by analysing infant mortality rates, injuries or communicable disease levels [2]. There is still huge concern in Europe about related issues such as widening inequalities, the rise of non-communicable diseases, problems of childhood obesity and poor mental health in children [2].

Health policy is an essential factor in improving health, because it embraces “courses of action (and inaction) that affect the set of institutions, organizations, services and funding arrangements of the health system” [3]. Despite this, there remains scant research on policy making in primary health care for children, and influencing forces and triggers.

Health is determined by factors outside as well as within the health system; meaning that various contextual elements must be taken into consideration when creating policies for health improvement. “Insufficient understanding of context and implementation ( …) contributes to the critical gap between research and practice” [4]. Context considered as the proximal and distal environment of child health policy “reflects a set of characteristics and circumstances that consist of active and unique factors, within which the implementation is embedded” [4]. Contextual factors that affect (health) policy refers to systemic factors [3]. They can be considered at political, economic and social level and might be analysed from both national and international perspective [3]. Leichter (1979) [3, 5] identified four overarching categories: cultural, structural, situational and international (exogenous) factors. Cultural factors refer amongst others to history, religion or ethnical aspects; structural factors are rather static parts of society such as political system, economy or demographic features; situational factors indicate temporal conditions of transient character; international factors point multilateral actions at global level [3].

To improve the quality of children’s health care in European countries it is therefore vital to understand how and what context influences policy making and the adoption of best practice.

This study is part of an EU Horizon 2020 project; Models of Child Health Appraised (MOCHA) which aims to identify and appraise primary child health care systems in Europe in 30 EU and EEA countries. MOCHA involves 19 institutional partners from 11 countries and integrates professionals in paediatric, adolescent and family practice medicine, child public health, nursing, psychology, policy and health management, political science, sociology, statistics, informatics, epidemiology and health economics [6]. The epidemiology of children’s health has shifted from a communicable-disease focused model to one where chronic illness, concerns with mental health and multiple morbidities are more common presentations. This shift requires the primary care systems to adapt accordingly, and MOCHA contributes to such solutions in the form of identifying and promoting optimal models of child health care in European context. This paper presents the in depth results of analysis of the contextual perspectives that are determinants of child health policy [7].

The main objective of the current study was to explore contextual determinants of child centric health policy, while taking into account the socio-cultural, structural, situational and international background.

## Methods

### Theory

Our methodological approach links the constructionist perspective described by Charmaz [8] and her data-driven inductive perspective with elements of deductive coding. This approach was adopted in response to the nature of the research group and to adjust to the MOCHA project’s overall methodology - MOCHA survey. Data were collected via 30 national experts in the field of children’s health care from European countries, each acting as the Country Agent (CA) for their country for the MOCHA project [9].

### Questionnaire. Development and distribution

The CAs, all of whom are competent English speakers and writers, were asked to identify the three most prominent public and professional discussions related to child services in their country in the preceding 5 years (2011–2016). They did this by answering a survey questionnaire, designed by the project researchers and created in Microsoft Word. The large geographic range of the project meant that using written questionnaires was the most practical way to obtain our data. The questionnaires consisted of a number of open-ended questions. These asked the CAs to specify the object of discussion; whether the child was considered directly or indirectly; the area of child health care to which the discussion was related (for example, school health, or preventive health care); the broader characteristics of the case; the level of discussion (such as whether it focused on individual experience, a community or a national issue); the characteristics of the vehicle of public expression; and the outcome of the case. This structure allowed the CA to provide detailed free text information, as well as providing enough structure to enable comparison between issues and between countries in the analysis.

The questionnaire was emailed to each CA by the project Research Coordinator. The respondents were given approximately 1 month in which to identify the ‘hot topics’ in their country, and return their written answers to the Research Coordinator. On receipt of the completed questionnaires, the Research Coordinator immediately passed them on to the researchers for analysis. Once the initial deadline of a month was over, the analysis of the questionnaires began, with any later replies being added to the analysis as they arrived. Regular communication between the Research Coordinator and the CAs and the researchers ensured that a good response rate was achieved.

### Analysis

The data collection was followed by the process of pre-reviewing the data and incorporating it into the software for qualitative analysis NVivo 11. During the coding process, which adhered to Charmaz [8] recommendations, we defined the content of the data and analysed the material phrase by phrase in order to transform the text into codes. Next stage was categorisation of our data understood as the analytical process of the certain codes selection due to their particular significance, and extracting common themes and patterns identified in many codes in order to create the analytical conception and define the properties of the extracted categories. Some codes were included in various categories and classified under different headings as categories were not mutually exclusive. Eventually we constructed the schemes of the identified processes and elements. The constant comparison approach understood as process of perpetual correlating new findings with those which has emerged previously was used at all stages of the research process.

In order to explore contextual determinants of child health policy across European countries, the results were synthesised based on the adapted classification of contextual determinants proposed by Leichter (1979) [3, 5]. The contextual determinants were identified from the analyses of the 71 resulting cases. Inductively identified codes were grouped into categories which we deductively assigned into the four groups determinants described by Leichter.

### Respondents

The data collection was carried out between June and December 2016. Representatives of 24 countries of the European Union (EU) and European Economic Area (EEA) responded to the question. Countries missing from our analysis were Belgium, Cyprus, Luxembourg, Sweden, Slovakia, Slovenia. In total, 71 cases were described, all of which characterised areas of public concern related to children’s primary health care in European countries.

## Results

**Fig. 1:**
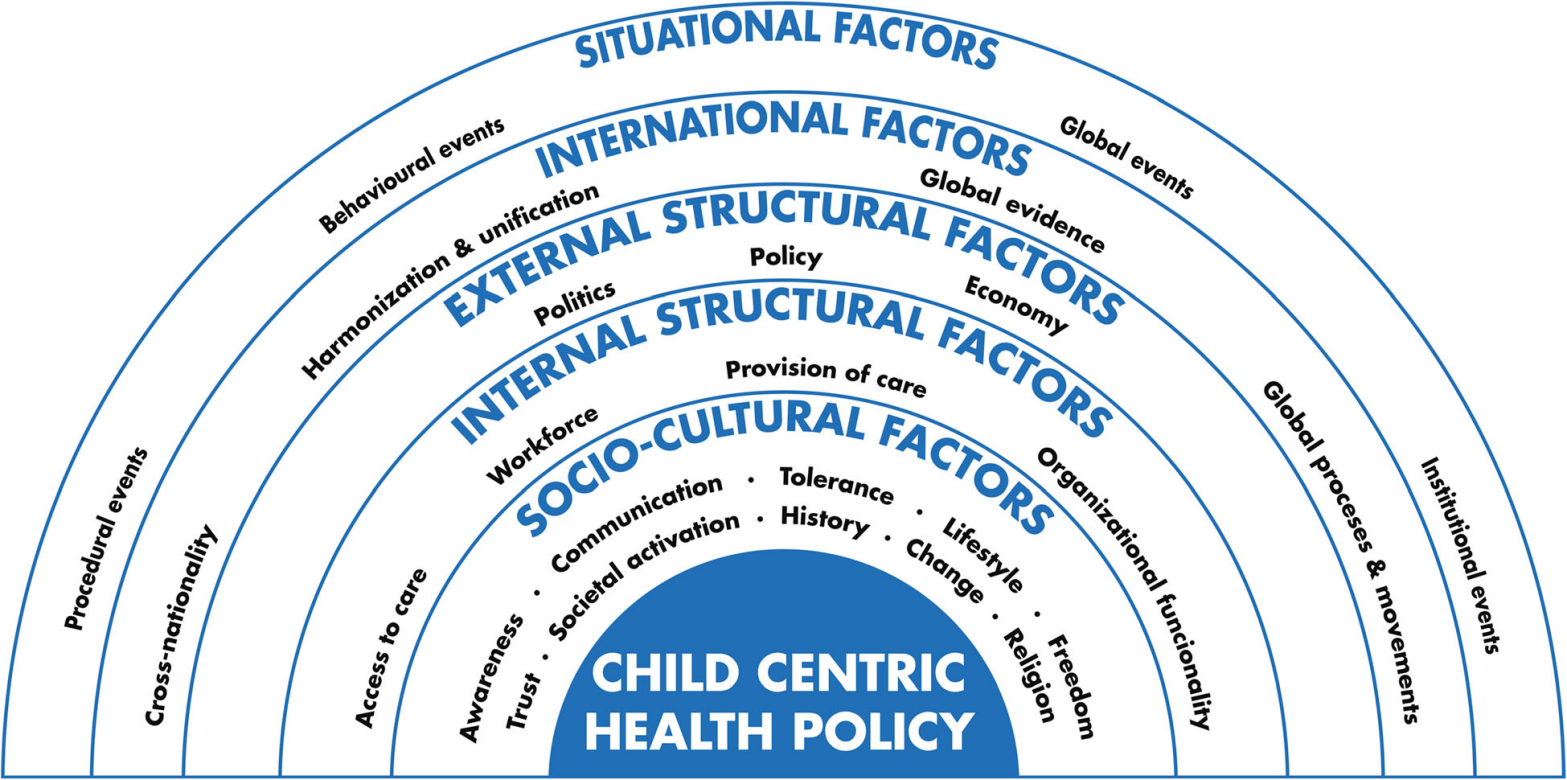
Contextual determinants of child centric health policy

Our goal was not quantitative, but to explore the determinants which seem to influence the way the children’s health policy reacted to issues, or is created across Europe. Figure 1 presents contextual determinants of child centric health policy which we identified during the research process. The findings are presented in four categories: Socio-cultural determinants; Structural determinants; International determinants and Situational aspects. Each category contains examples from the CAs of the major issues that have emerged in the past 5 years. An overview of the case studies can be found Table 1.

**Table 1:** List of cases by country with the information on MOCHA Agreed Primary Care Lead Practitioner (Primary paediatrician lead, GP lead, and mixed: combined with GP and Paediatrician or other medical staff) [10]

Case	Country
Lifestyle changes obesity and overweight	Austria (MIXED)
	Iceland (GP)
	Malta (GP)
	Netherlands (GP)
	Norway (MIXED)
	Portugal (MIXED)
	UK (GP)
Lifestyle – nutrition	Italy (MIXED)
	Poland (MIXED)
Lifestyle – smoking	Latvia (GP)
Mother health	Latvia (GP)
Mental health	Denmark (MIXED)
	Germany (Paediatrician)
	Greece (Paediatrician)
	Latvia (GP)
	Portugal (MIXED)
Environmental health	Italy (MIXED)
Medicalization	Denmark (MIXED)
	Iceland (GP)
Vaccination	Bulgaria (GP)
	Croatia (Paediatrician)
	Czech Republic (Paediatrician)
	Denmark (MIXED)
	Estonia (GP)
	France (MIXED)
	Greece (Paediatrician)
	Italy (MIXED)
	Lithuania (MIXED)
	Romania (GP)
	Spain (Paediatrician)
Child poverty	Greece (Paediatrician)
	Ireland (GP)
	Malta (GP)
	Portugal (MIXED)
	Spain (Paediatrician)
Child abuse	Croatia (Paediatrician)
	Iceland (GP)
	Netherlands (GP)
	Norway (MIXED)
	UK (GP)
Children rights	Denmark (MIXED)
	Finland (MIXED)
	France (MIXED)
	Romania (GP)
Children rights - Discrimination of child with chronic condition/vulnerable child	Austria (MIXED)
	Bulgaria (GP)
	Croatia (Paediatrician)
	Czech Republic (Paediatrician)
	Poland (MIXED)
System organization	Czech Republic (Paediatrician)
	Finland (MIXED)
	Hungary (MIXED)
	Poland (MIXED)
Access to care	Malta (GP)
	Norway (MIXED)
	Finland (MIXED)
	UK (GP)
Provision of services	Austria (MIXED)
	Bulgaria (GP)
	Estonia (GP)
	Iceland (GP)
	Ireland (GP)
	Lithuania (MIXED)
	Netherlands (GP)
	Romania (GP)

### Socio-cultural determinants

**Fig. 2:**
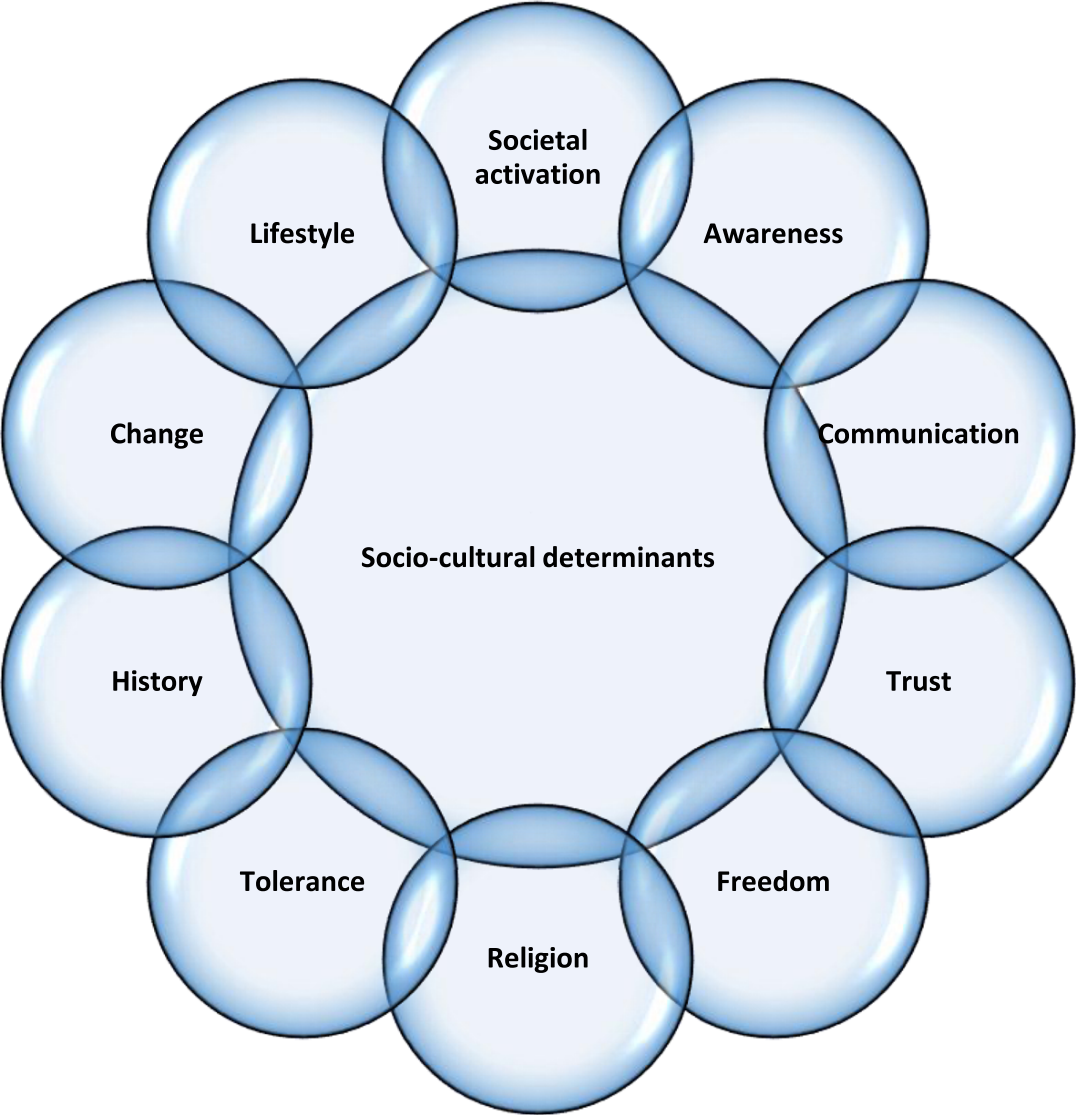
Socio-cultural determinants of child centric health policy

Several key socio-cultural characteristics defining national issues were reported. These were grouped into categories of societal activation; awareness; communication and trust; freedom; tolerance; religion; history; change; and lifestyle. These are illustrated in Fig. 2.

#### Societal activation

Examples of societal activation were in terms of public activism and individual activism. In the Czech Republic, societal activation took the form of a mass initiative to advocate for freedom of choice regarding vaccinations, which was largely influenced by anti-vaccination sentiment.

“*The public debate led (…) to issues related to ‘adverse effects‘ of vaccination and finally (on the level of the Czech jurisdiction) to the issue of responsibility and financial compensation for adverse effects of mandatory vaccination*” (Czech CA).

In Austria, public discussion emerged as a result of the experience of the parents of a chronically ill child. They had actively managed their child’s condition, but faced considerable challenges in the school environment. The educational authorities feared applying inappropriate procedural steps to accommodate the child, who was consequently refused school entry. This highlighted the general lack of adequate support for children with special needs in the school system (Austrian CA).

#### Awareness

Awareness of a problem or of its impact was demonstrated by the issue of child poverty and its impact on well-being, as described by the Spanish CA. Awareness was also linked with access and availability of information as reported by the Austrian CA. Lack of knowledge about the risks and consequences of a disease was one of the determinants of a crisis of vaccine hesitancy and vaccine rejection in France. An epidemic outbreak of 026: H11 E Coli was blamed on a supposed link to a vaccination in Romania which:

“*created a sort of a hysteria outbreak ( … ). Many parents/religious groups militated for non-vaccination after this situation, claiming a relation between vaccination of the child and symptoms of [the disease - haemolytic-uremic syndrome]. The investigation that followed showed no association, as does the literature ( … ). The Ministry of health gave a press declaration regarding the lack of any association between vaccination and the death of the babies*” (Romanian CA).

#### Communication and trust

The epidemic outbreak in Romania and a lack of clarity in terms of informing the public about the progress and results of investigations intensified a problem of lack of trust in the competent authorities and simultaneously reinforced a crisis in social capital. The lack of direct communication between hospitals, primary health care, schools and social services in Norway was seen to reflect the apathy of the social environment to child abuse, which resulted in the death of an 8-year old boy. This incident highlighted the issue of a deepening lack of trust expressed by a decreasing confidence in all institutions. This was also reported in terms of a growing vaccine hesitancy, for example in Italy where:

“*for several decades there have been organisations and groups opposed to vaccines that promote campaigns against vaccines, urging parents not only to refuse MMR (which is optional) but also the mandatory vaccinations. The percentage of people in Italy vaccinated against mumps, rubella and measles decreased between 2010 and 2012 from 90.5 percent to 89.2 percent; that is about six thousand vaccinations less. Adverse rulings could lead to further distrust of an essential tool such as vaccinations to monitor infectious diseases*” (Italian CA).

#### Freedom

The national debates about vaccination often are accompanied by the issue of freedom expressed by respect for the rights of children; for example the right to refuse mandatory vaccination without the risk of financial sanctions or limited access to the services (the Czech Republic and Lithuania). The right of access to healthcare was discussed broadly in Ireland, which was prompted by a change in the eligibility criteria for Discretionary Medical Cards (which enable access to free care) which was discussed.

“*The issue provoked debate about the rights of children to have access to healthcare. It was felt that health policy which targeted children, and particularly those with long-term illnesses or complex healthcare needs, was not appropriate and was fundamentally wrong*” (Irish CA).

#### Religion

In accordance with French CA heated debate referred to contraception for adolescent girls, in particular to access to emergency contraceptive pills. Discussion surrounded the intention of pharmacists to incorporate a conscience clause context within their code of ethics. In France, the introduction of a new law on marriage for all, which was also called the Taubira law:

“*opened new rights for marriage, adoption, and inheritance in the name of equality and shared freedoms”* (French CA).

As a result of this new law, it was felt in France that the concept of family changed, and this then influenced traditional concepts which were based on religious and conservative values.

*“This strengthened the political influence of the proponents of these values, which for many of them are also against abortion and contraception”* (French CA).

Within recent years in Malta, the problem of child poverty has become an issue of public concern. In Malta, the issue has become interwoven with religion and with tradition; as poverty levels were perceived to have increased because of marriage breakdown as a result of the introduction of legal divorce in the country. This, combined with increasing numbers of immigrants living in the country meant that many felt:

“*traditional socio-cultural milieu associated with the Roman Catholic religion and the upholding of the family as a traditional value has been eroded. As a result, more and more children are living in single-parent households and these are at the highest risk of poverty*” (Maltese CA).

#### Tolerance

Amongst the issues analysed and discussed in Europe, many reflected contemporary socio-cultural dilemmas. Migration and the changes in the traditional family pattern brought about the emergence of discourse of tolerance. In Finland, for example, the sexual education programme has been changed to include homosexuality, which has prompted opposition from conservative minority religious groups. As part of this discussion:

“*[the notion of] children’s rights have been used to oppose the gender-neutral marriage, even though the main focus has been on being against sexual and gender minorities and their rights*” (Finnish CA).

Finland also reported the issue of asylum seekers, particularly that of minors who seek asylum without their parents or guardians. There was public debate both for and against migration, from the need to provide all the required services on one side to the potential health threats facing Finnish society from immigrants on the other.

“*Regarding children, the very unwelcomed focus has been on the small number of adults who claimed to be minors. Also, infectious diseases ( … ) have been discussed from the point of view that the asylum seekers would cause a threat to the Finnish population. [ … ] The political discussion was divided into ‘migrant critical’ and ‘tolerant‘ groups. In the name of free speech, even racist and xenophobic claims should be accepted*” (Finnish CA).

The problem of unaccompanied asylum seekers was also discussed in the United Kingdom.

“*While there is a clear concern for the wellbeing of these children, the current political climate is less favourable toward immigration - captured by the anti-immigrant rhetoric spouted by pro-Brexit campaigners in the UK and the growth of far-right parties across Europe. Since Brexit, there has been an increase in the reported number of hate crimes suggesting that there is an anti-immigrant sentiment within the UK which is not positive for those who are advocating for greater care for UASCs [unaccompanied asylum seekers]*” (United Kingdom CA).

#### History

Tradition is closely connected to a country’s history, which as a result was extracted as a separate category from the data. As in most of these cases, the impact of past policies and inherited traditions. For example, archaic policies were linked to the child abuse scandal in Iceland.

“*Many of the historical cases have roots in a policy of taking children into custody who were deemed to fare badly in their home environment. This policy has been changed*” (Iceland CA).

Historical factors were also associated with the debate about the system of care for vulnerable children in the Czech Republic. The issue was linked to the adoption of a:

"*National Strategy for Protection of Children's Rights - the right to a childhood, which implies the activity of unification of conditions for the operation of services*" (Czech Republic CA).

The Czech Country Agent reports:

“*The case of care for vulnerable children (including family / foster family / institutional care) and inclusive education (was) strongly linked to the nationwide debate about advantages/disadvantages of the inherited “socialist-time” care, where such children were generally provided care in specialized institutions (the so-called “special schools” or “practical schools”). It also to a certain degree addresses the issue of “inclusion” of the Roma children and adolescents. The proposed change requests the change of beliefs (that compromised children are “better off” when cared for in cohorts with similar children)”* (Czech Republic CA).

The former socialist practices of organising care for children in residential units were seen as important influencing factors in the issue of overmedication and prescription of medication for long periods to children in Romania. The children’s medication regimes were rarely revised, and there was very little provision of psychotherapy or other forms of therapy.

“*The case has historic roots, as children were in state care during the socialist period and even later, after the political shift in 1989 were neglected and abused in large residential centres. Till today, residential units for children in care are under-staffed, with existing staff badly paid, with few specialised professionals; children do not benefit from rehabilitation or therapy, even when they are diagnosed with post-traumatic stress disorder, ADHD, disabilities or other psychiatric disorders*” (Romanian CA).

When the rights of children with disabilities and their caregivers were discussed in Croatia the parents of children with disabilities protested against discriminatory, contradictory and inequitable laws which were changed to their disadvantage. This conflict had also strong historical correlations because the Croatian:

“*socialist background which fosters strong social protection and is characterised by equality commitment*” (Croatian CA)

provoked the confrontation of the negative changes in social protection rights with parents’ and caregivers’ disapproval. The issue was especially sensitive as the children with disabilities were considered a vulnerable population group.

The unique model of primary care medical education in the Czech Republic is part of a historical tradition. In the Czech Republic, there exists the registering general practitioner for children and adolescents in primary care; which is a different speciality to that of the primary care paediatrician and that of the general practitioner (PLDD). Proposed legislative changes to medical education would cease to recognise this role, and met with general disapproval from the Czech medical profession.

“*Ongoing efforts of paediatricians to merge into the discipline of child medicine and resistance of PLDDs underpinned by the fact of so far existing differences in training and preparation for functioning as PLDDs. So far, existing "uniqueness of this field of specialisation" prevents the migration of physicians into this field. The rivalry between disciplines of PLDD and Paediatrics (child medicine) for long-time employment of paediatricians exists in hospitals*” (Czech Republic CA).

#### Change

Contextual change involves shifts in the proximal and/or distal child environment or it might be a phenomenon at the macro or micro level. Several examples were found to correlate with contextual change, including changes to an early intervention model in Austria. An outreach Service “Frühe Hilfen” was implemented to improve support and opportunities for children and parents by:

“*support mainly for vulnerable pregnant women, mothers as well as parents to cope with challenges in early childhood (0-6 years)*”(Austrian CA).

Frühe Hilfen aims to meet its objectives:

“*through the cooperation of professionals and institutions in a region and the visualisation and networking of existing offers of help. So-called family companions support families in their home environment. They guide them through the social and health system*” (Austrian CA).

Contextual change is increasingly linked to the modernization of everyday life, such as the concern about childhood obesity. In Portugal, changes in living conditions in the last four decades have been blamed for the increased prevalence of childhood obesity.

“*The improvement in living conditions has allowed families to acquire goods that foster passive transportation (e.g. cars). Furthermore, the eating habits of the population have been changing; people have left the Mediterranean diet and the consumption of hypercaloric nutrients has been increasing*” (Portuguese CA).

New family structure and subsequent parenting problems have been related to violence in schools, including bullying, in Latvia. Changes in parenting norms, which accompanied the introduction of legalisation to enable home births in Estonia contributed to an overall increase in the number of home births compared to the 1990s and early 2000s. This demonstrates not only the shift in parental attitudes towards delivery and parenthood, but may be also interpreted as a de-medicalization of motherhood and childbirth. Children’s health and experience may also be affected by industrial factors, such as the advent of the 4.0 Industry [11] and automation, digital media and its impact on the mental health of children and adolescents is a topic of concern in Germany (German CA).

#### Lifestyle

Digital media and its use in schools is a component of modern lifestyle interpreted as a set of behaviours which may, directly or indirectly, affect positively or negatively children’s health. Lifestyle is a component to take into consideration while defining child health policy priorities. In the United Kingdom, discussions about childhood obesity and its relationship to diet and exercise is an example of lifestyle factors.

“*On a UK level, The Obesity Alliance has called for restrictions on advertising before the 9pm watershed for food and drink products that are high in saturated fat, salt and sugar, investment in active travel, action on reformulation of food and drink high in fat, sugar and salt and a tax on sugar-sweetened beverages. In 2013 the Welsh Assembly passed the Active Travel (Wales) Act, which is hoped will boost travel on foot or bicycle rather than by car*” (UK CA).

A debate on the school environment and how it appears to foster obesity and overweight is of high importance in the United Kingdom.

“*In England, the risk of being overweight or obese increases as children progress through primary school. In Reception class, around 1 in 5 children are overweight or obese; rising to around a third of children by the time they reach age 10 or 11*” (UK CA).

The problem of obesity and lifestyle-related issues was also reported in Austria, where it was stressed that there was a shift in health oriented behaviours. Children and adolescents often sit for several hours a day in front of a screen. In the discussion on the increased “school autonomy”, the decreasing amount of time for physical activity at school and the high prices for healthy food were prominent factors. In Iceland, a sedentary lifestyle, computer usage and the popularity of social media were identified as important socio-cultural factors which affect obesity rates. The debate focused on easy access to unhealthy products and a lack of an effective food pricing policy. In Portugal, the argument focused on the lack of active transportation (such as walking or cycling) to school. In contrast to the discussions about lifestyle mostly in terms of obesity and overweight, new dietary trends and alternative nutritional habits are also the focus of lifestyle debates. What was termed the healthism movement in Italy referred to a parents’ vegan diet (which) impacted on the health of their child, such a diet:

“*follows a healthy regime, [but] it lacks food values such as proteins which, in adulthood can be replaced with plant-sourced food. Such a diet can, however, be detrimental to a child, if not correctly followed by doctors, as they need a more varied diet and a proper balance of supplements, vitamins, and proteins*” (Italian CA).

Those arguments were part of a very vigorous and sometimes aggressive debate between supporters of special diets, and members of the official dietary science organisations; which grew to a public discussion on animal protein, lactose, gluten and so on.

### Structural determinants

**Fig. 3:**
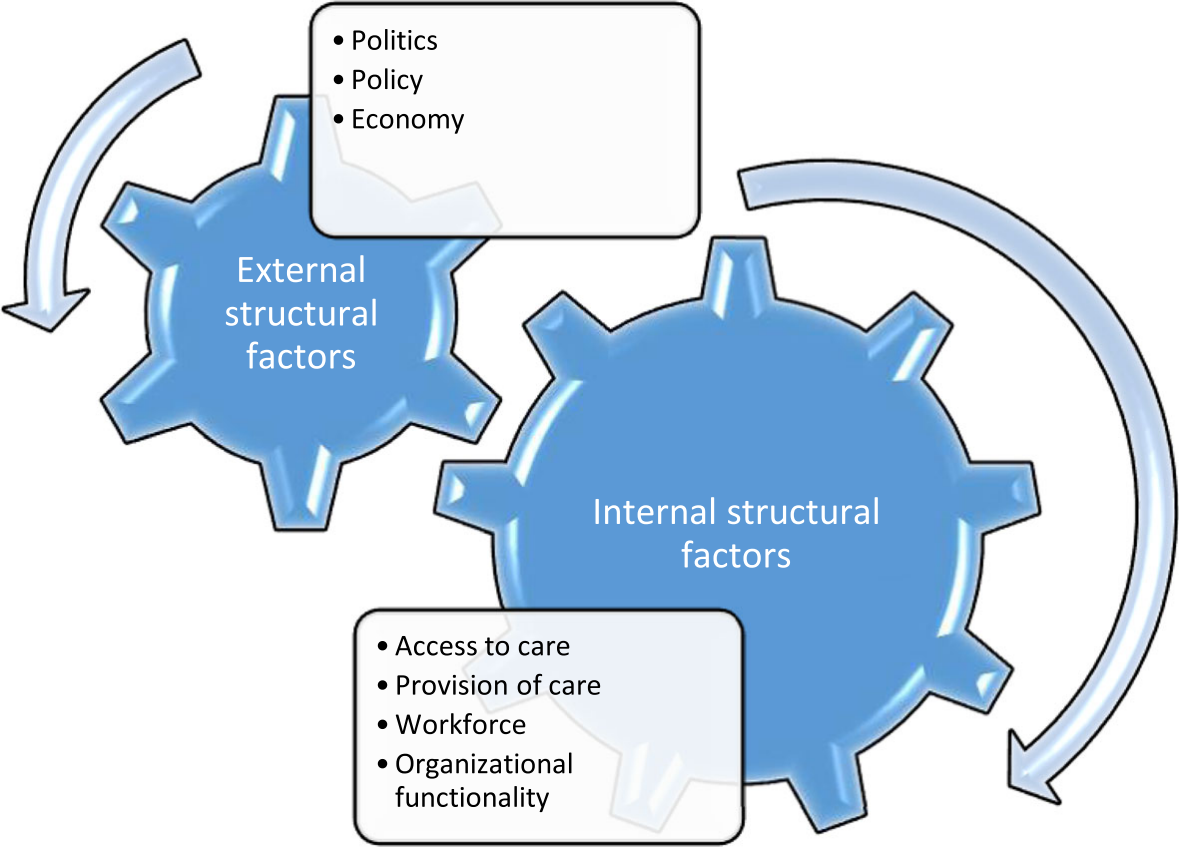
Structural determinants of child centric health policy

Some of the determinants of children’s health policy can be classed as structural. This means that proximal and distal elements of the children’s health care system influence the way the services are provided. As our research focuses on children’s health, the relationship of the health care system with other systemic elements remains crucial. We have defined the structural factors as internal and external determinants. The internal determinants are those identified within the structure of health care and policy such as access to care, provision or care, workforce issues and organizational functionality. We consider them as those which remain in direct relationship with the health care system. The external determinants such as policy, politics and economy, relate to the elements indirectly correlated with health care and policy in accordance with the approach “health in all policies”. This is shown in Fig. 3.

### Internal structural determinants

Our data, which established the structure of internal determinants, was comprised of interdependent processes such as access to care and provision of care. It highlights also the issues of organisational culture, workforce, and organisational functionality of the system.

#### Access to care

Access to care as the contextual determinant of children’s health care and policy was identified by Norway in the case of:

“*A ten-month-old boy ( … ) died on the way to the central hospital in an air ambulance. Living in the north-western part of the country, in a place with a small hospital where the paediatric ward is closed at weekends. ( … ) No air ambulance available before late in the afternoon from a regional hospital. On the way to the hospital, the surveillance system in the air-ambulance failed ( … )”* (Norwegian CA).

This case initiated a national discussion about the wider context of hospital localisation and access to care. The issue of a policy for:

“*centralisation of services to ensure a large enough volume of care to provide care of a suitable quality” became part of this discourse. The indirect relationship with health care provision referred to the “conflict between the relationship and independence and responsibility between the local level hospital, hospital trust level, regional health authority level and national level (Minister of health*)” (Norwegian CA).

A further incident related to access prompted national debate in Norway, when the regional health authorities proposed the closure of neonatal wards for children born before the 26th week of gestation. This provoked an outcry at the local level; despite a background debate between the strong tradition of independence in the regions and recognition that although access to obstetric services was available, these were often of questionable quality,

Public concern in terms of access to specialist care and medication was raised in Iceland in relation to concern about over-medicalization. A gradual increase in the use of methylphenidate for ADHD was observed. The Icelandic CA stated that:

“*the controversy lies in if this increase in use is medicalization of normal behaviour of children or a new phenomenon in child development or a problem that has hitherto been unrecognised”. *He stressed that, contextually,* the issue was linked with good access to child psychiatrists and gradually-improved access within secondary primary healthcare to diagnostic services for ADHD, including psychologists*" (Icelandic CA).

In Greece, a controversy related to child worsening mental health was mirrored by the hardship of the economic situation. The concern was raised by the health professionals as mental health issues in children were identified as a consequence of the crisis. In contrast to the situation in Iceland, the problem of access to care was:

“*linked to health reform and austerity measures, which have reduced the number of available services, the amount of state subsidy for these services, the number of professionals such as special education assistants, “shadow” teachers etc*.” (Greek CA).

In Latvia, the problem of high maternal and perinatal mortality rates was raised. This was linked to issues of accessibility of health care and regional restructuration of perinatal health care. Many maternity beds in local hospitals had closed, and there had been greater concentration of health care at the regional level to improve service quality. A lack of health care professionals was identified as a problem, especially in rural regions.

#### Provision of care

The issues of access to care interrelate to provision of care. In Estonia, public concern was raised after the:

“*development plan proposal of the Estonian Health Insurance Fund for 2017-2020, does not include the provision of paediatric services in county hospitals for cost-reduction purposes, which means that paediatric services will only be available in four major Estonian hospitals*” (Estonian CA).

This was met with disapproval from the Estonian professional paediatric associations. This change was linked to the implementation of a new regional development strategy for 2014–2020 which aimed to improve the availability of services. In accordance with the document, some services including:

“*specialist medical care do not have to be available near a person’s place of residence, while services that link to basic needs such as primary education, police services, and primary health care must be available in all active regions across Estonia. To ensure that all citizens have access to services that are available only in regional centres, further development of transport links and infrastructure must be carried out*” (Estonian CA).

The proposed solution can be interpreted in terms of reorganisation of priorities and services provision with the goal of cost reduction and improved quality.

The contextual baseline for the Finnish health care system reform was also linked with a debate about the risks of limiting access to service provision. In the proposal, more than 300 municipalities, which currently govern health and social welfare services, will be merged into 18 regions.

“*It has been discussed how maternity and child welfare clinics, as well as school health care, will be placed in this new system, which aims to enlarge entities and centralisation. The discussion was linked to economic crises and the need to downsize the public health services ( … ) after the recently ended long-term recession. The counter-discussion was on saving for our future, as children are our future*” (Finnish CA).

In Ireland, provision of care was indirectly linked to issues of restructuring as evidenced by the centralisation of decision making about service provision, via the scheme of Discretionary Medical Cards (which give entitlement to free health care). Discussion about the revision of the eligibility criteria for Discretionary Medical Cards was linked to centralisation of the process to determine eligibility for free health care (and) led to a significant public debate about how the State should support children with complex or long-term healthcare needs.

“*This was associated with a significant geographical variation in terms of eligibility criteria, particularly so for Discretionary Medical Cards ( … ). In 2011, the management of Medical Cards was changed to be centrally managed by the HSE Primary Care Reimbursement Service (PCRS) in order to streamline the service, reduce variability, improve work processes and improve transparency*” (Irish CA).

#### Workforce

The importance of workforce issues were also underlined in our data. In the Czech Republic, the debate about the modification of the proposed system of post-graduate care for medical doctors was strongly linked to the large number of paediatricians; the high proportion of doctors aged 55–75 years; the risk of a drain of healthcare professionals to other countries; and the debate about remuneration of the medical professions. In Latvia, the problem of workforce shortages was identified as an important factor in high maternal and perinatal mortality rates.

A nurses’ strike in the Child Health Centre in Warsaw (the largest specialist children’s hospital in Poland), highlighted workforce conditions, including the insufficient number of medical personnel and the low wages of Polish nurses.

“*Poor working conditions of nurses are related to small number of nurses in Poland, compared to the needs of patients. ( … ) One of the demands of the protesting Child Health Centre nurses was to increase the number of nursing staff because the long work hours seriously burdens them physically and mentally, and creates a risk for their patients”.* (Polish CA).

In Romania, a fire in an intensive therapy ward for premature babies highlighted shortcomings in workforce. There was no member of the medical staff in the ward at the time of the fire. This case was linked to the problem of:

“*questionable political decisions that affected the normal functioning of the health services. One of these decisions was referring to the cessation for an extended period of any employment in all public services, including the health system which was already facing an important reduction in personnel. The risks for the functioning of the intensive care for premature infants was ignored; the hospital services functioned with the same capacity, but much-reduced staffing, without procedures or regulations of what to do in case of a nurse being alone and she needs to leave the ward*” (Romanian CA).

The problem of a lack of staff on duty in intensive care for premature infants was highly politicised by being associated with the severe austerity policy, began by the former Government, and the political party that sustained that government.

#### Organisational functionality

The Romanian case introduced another contextual factor which was coded as the lack of an organisational culture as a symptom of the organisational (dys) functionality of the system. In the Romanian case, weak leadership and a lack of technical maintenance procedures were blamed. This was accompanied by a lack of respect for safety procedures, including shortages of appropriate equipment. Additionally:

“*the medical leadership did not stand up against the political ruling which aggravated the medical personnel shortage so much that it clearly endangered patients’ lives*” (Romania CA).

This situation brought about the discussion about the capability of the Romanian emergency system to respond to emergencies, in terms of basic equipment, workforce issues as well as inappropriate procedures and protocols. This provoked longstanding debates on the need to invest more in the health system and correct negligence, inappropriate treatment of patients, insufficient staffing and inequalities in health care. The arguments on the (dys) functionality of the Romanian health care system were intensified after the:

“*series of situations where the medical system was put on the spot for being understaffed, not well equipped, and not responsive enough to the suffering of patients. The last case was the death of 58 young people in an underground club fire, in Bucharest, the capital, where besides the club itself, the medical system was accused of a low capability to react in the case of disaster that might endanger many lives; one of the issues was the presumed dishonesty of spokesmen for the health authorities*” (Romanian CA).

Cases such as the fire in the Romanian hospital, the overmedicating of children in residential care discussed earlier can be seen as examples of poor organisation and governance of the health care system.

The inefficiency of a health system might be observed in the example of the Polish nurses’ strike. The Polish CA stressed that:

“*the strike was local and intrastate and is discussed in the context of the overall dysfunctionality of Polish health services. It should be emphasised that, not coincidentally, the strike took place in an institution dealing with specialised, difficult cases: the method of funding procedures drives the hospitals into debt, which means that hospital managers have a limited ability to raise wages and hire new staff. So, according to both hospital management and governing party politicians, the problem is not incidental but results from years of neglect of reforming the entire health service in Poland”.*

The Polish CA states that the Polish health service is in a state of permanent reform, which increases organisational chaos and hinders comprehensive and systemic changes in the situation of medical personnel (Polish CA).

### External structural determinants

Child health policy is not created in isolation. There are external determinants circulating around health care, which directly and indirectly affect the way health policy is formulated or health care is provided. These determinants may influence the hierarchy of priorities, in terms of the position of child health amongst other values. External structural determinants are interpreted as those factors on a macro level which influence the way problems are solved, and issues are negotiated in the area of children’s health care. Our data reveal a strong group of determinants in the field such as politics, policy and economic and financial factors.

#### Politics

Children’s health services are contextually influenced by political initiatives. In Estonia, the formation of a new government affected the discussion on mandatory vaccination and its potential links with national family benefits. In the Estonian coalition agreement from 2015, a clause linking the disbursement of family benefits to periodic visits to the family physician was included. It met with strong approval from an Estonian family physician Marje Oona, who claimed in a newspaper article that politicians should definitely consider doing so.

“*The case was linked to the agreement of the Estonian Reform Party, Social Democratic Party and Pro Patria and the Res Publica Union on the formation of a government and on the general principles of the action program of the government coalition. This document was the foundation of which the above mentioned family physician stated that linking family benefits with vaccinations is an idea worth considering*” (Estonian CA).

The introduction of changes to the post graduating system for medical doctors in the Czech Republic described earlier, was as a consequence of election promises.

“*A more simple, transparent and functional system of professional medical education is basically the election promise of the current governmental coalition in the Czech Republic. Professional organisations (including a Czech medical chamber, medical Society of J.E. Purkyně and Association of GPs for children and adolescents) have so far not arrived at a satisfactory consensus with the policy makers and officials of the Ministry of Health and the resulting solution remains the responsibility of the Parliament of the Czech Republic*” (Czech Republic CA).

Political involvement was also observed in some cases directly related to problems of children’s health services which were commonly recognised as important. A kind of “political awareness” was observed in cases linked with globally-prioritised issues. In Malta, for example, obesity has been on the political agenda for some years and:

“*more recently the opposition put forward a bill on obesity. This was eventually modified as a legislative act on Non-Communicable Diseases known as the Healthy Lifestyle Promotion and the Care of Non-Communicable Diseases Act which was passed unanimously in Parliament*” (Maltese CA).

The health of vulnerable children has been present in political discussion in the Czech Republic since 2009. In September 2016 the “inclusive school system” was introduced. Conversely, a lack of “political awareness” of the problem of child inequalities moved public sentiment in Poland. Some parents of disabled children manifested their disapproval about a lack of adequate support for people with disabilities. It was claimed that the prior cause of such was the lack of political will and the financial deficit in the Polish budget (Polish CA).

#### Policy

The level of political awareness is expressed by the actions undertaken at policy level. This is expressed by implemented legal solutions. In Latvia, discussion about reducing children’s exposure to passive smoking was accompanied by amendments to the law on “Tobacco products, herbal smoking products, electronic smoking devices and the fluid circulation law” in order to harmonise with European requirements, included in this policy discussion were the issues of relatively low prices and easy access to cigarettes.

In Poland, the government, introducing changes in the act on food safety and nutrition, claimed that:

“*There is no doubt that the emergence of overweight people and obesity, especially among children and young people, is a problem that requires urgent action to effectively eliminate their causes. It is necessary to strengthen policies in the field of public health, in particular, health promotion, conducive to enhancing the knowledge and skills of consumers, enabling them to make healthy choices in nutrition. A risk factor for non-communicable diseases which are currently the greatest burden of the population is an unhealthy lifestyle, including nutrition*” (Polish CA).

In Croatia, changes in laws and ordinances regarding children with disabilities and their parents’ rights provoked numerous protests. Parents claimed that the law was changed to their disadvantage. Non-profit organisations for the promotion of welfare entitlements for children with disabilities raised objections and the national Ombudsman expressed negative attitudes towards the government decisions (Croatian CA).

The implementation of the new vaccination scheme in Spain, caused fears of increasing social exclusion and lessening the vaccination rate for marginalised populations such as minority groups.

In Greece**,** the introduction of austerity policies and decisions about health reform, "have reduced the number of available services, the amount of state subsidy for these services, the number of professionals such us special education assistants, “shadow” teachers etc." (Greek CA). This has been linked to worsening child mental health in the country.

#### Economy

We also observed that economic and financial factors had an important impact on children’s health policies. Often, political and economic issues serve to mutually intensify the effects of each other. Austerity policies in Greece or Spain were the consequence of the economic crisis; which has in turn heavily impacted people from lower socio-economic classes. In Greece*,* in particular, mounting unemployment has caused intense social problems across the country. Often families have no income due to unemployment of both parents and rely solely on welfare, which is under reform because of austerity measures and is already limited. This situation contributed to worsening poverty and economic inequality affecting mostly the unemployed, single parent families and non-EU migrants and deepened the problem of child poverty and hunger.

When the new vaccination scheme was proposed in Spain, it was observed that public spending cuts were prioritised and less importance was given to the epidemiological and public health issues. Spending cuts in all public services contributed to the problem of child poverty. Particular attention was given to banks’ bailout costs and health care spending cuts.

In particular, the recession affected marginalised groups more severely, and families were placed at risk of social exclusion.

Severe consequences of economic recession and its impact on children’s health care and policy were also reported in Portugal. The crisis in the background contributed to both child mental health and child poverty. As a consequence of austerity policies, some families lost social support which significantly affected their living conditions. Additionally, the mental health of adolescents has worsened.

*“The effects of the crisis upon Portugal can be observed in several areas of the society. However, it was not expected that the crisis would affect so deeply many households with many children and adolescents. The HBSC repots in Portugal highlighted that mental distress was one of the most affected areas, children and adolescents feel less well, report a lower life satisfaction, more psychological and physical symptoms of distress and a lower hope in the future, and lower expectation towards the future”* (Portuguese CA).

The system deficiencies in Romania, illustrated by the example of the fire in the Obstetrics and Gynaecology Clinical Hospital, were linked to the severe austerity policy. Staff shortages were the consequence of political decisions such as the cessation of employment in all public services, including the health system for an extended period of time.

“*Given that the nurse was the only staff on duty in this intensive care for premature infants, the media soon identified and blamed the lack of staff as being responsible for the tragic event*” (Romanian CA).

Ireland was also affected by the global economic situation. The severe consequences were expressed by an increase in homelessness and the number of children and families living in emergency accommodation. Such a situation is related to the:

“*interplay between the economic recession, which started in 2008, and the resultant negative impact on income, employment, and the contraction of the construction industry which contributed to the current housing shortage*” (Irish CA).

The consequence of the economic recession led to several years of austerity measures; including pay cuts and increased unemployment. This contributed to a significant decrease in quality of life and health impairment. The revision of the eligibility criteria for Discretionary Medical Cards (for free access to health care) and centralization of the procedural steps was also the consequence of the economic downturn.

“*Centralisation of the Medical Card process was influenced by a need to improve efficiencies and cost-effectiveness, and also to reduce excess spending on the scheme*” (Irish CA).

The Irish CA reported that many of those who were refused Discretionary Medical Cards were refused on the grounds that the child’s condition did not cause undue financial hardship for the family, suggesting that one or both parents may have been working with an income which exceeded the threshold. However, because many of the families who spoke publicly about their experiences were in employment and were tax-payers, there was a sense that this was ‘one cut too many for middle-income earners (Irish CA). The societal reaction came just in time to restore 13,000 Discretionary Medical Cards as an interim measure.

Lithuanian concerns about the appropriate functioning and access to a Child Development Centre for children with developmental and behavioural disorders, was linked to political negligence and limited financial resources.

“*In recent years, there has been more than a half a million drop in governmental funding of the Child Development Centre as for all of the paediatric health care*” (Lithuanian CA).

In Austria, children diagnosed with epilepsy, diabetes, asthma, or cystic fibrosis have more difficult access to schools and/or kindergartens than healthy children. This has been explained by a fear of caring for a sick child, and a lack of the necessary resources to provide effective care As a result of the national debate, the organisation and financing of care were switched to parents.

### International determinants

The global situation and global processes are reflected by international factors. They are related to the phenomena presented at macro level. In particular, respondents mentioned the significance of the migration wave (Finland, the UK) or the economic crisis (Spain, Portugal, Malta and Greece). Additionally, the intensity of the global movements was stressed as well in the case of obesity prevalence at a global level as well as anti/pro-vaccination movements across Europe. The international context is not insignificant for children’s health care policy. Membership of regional and global organisations facilitates diffusion of information and exchange of knowledge, but also obligates a respect of shared values and the adoption of commonly-agreed rules.

#### Global evidence

Global reports and comparison studies were identified by many Country Agents as an important source of information which provoked or supported the national discussion about an existing problem.

An important source of the data was the Health Behaviour in School-Aged Children (HBSC study and other World Health Organisation (WHO) analyses, which are important sources of information about children’s health. The WHO data was reported as the international determinant which affected the debate on obesity in the Netherlands, as it was claimed that:

“*the discussion about childhood obesity isn’t a national discussion, but a global discussion. The WHO has published numbers about this topic*” (Dutch CA).

Similarly, the obesity issues debated in Portugal used the evidence-based arguments published from the HBSC study.

In Austria, when the problem of a lack of sufficient physical activity at school correlated with obesity, prevalence was discussed with strong reference to statistical data. In the last case, the OECD reports from 2010/2011 provoked the Austrian Health Ministry to establish some measures to promote healthy snacks. Currently, the initiatives are slowing down due to a lack of appropriate financial support. However, the UK reported that childhood obesity was considered as an international issue. By reference to global evidence, the WHO Report on Ending Childhood Obesity 2016, it was emphasised there was a strong need for:

“*coordinated cross-sectorial action and a strong focus on actions in pregnancy and early life*” (UK CA).

Latvia introduced changes in maternal mortality prevention after taking into account the data reported by the WHO.

*“The maternal mortality audit system in Latvia has been developed by the support of The Ministry of Health of the Republic of Latvia. It was organised with WHO-supported training for specialists in 2012 and 2013*” (Latvian CA).

The problem of bullying in Latvia was reported in a HBSC study, where it was shown that:

“*an average 14% of Latvian 11, 13 and 15-year-old pupils have suffered from bullying at least 2-3 times in recent months and around 7% of Latvian 11, 13 and 15-year-old pupils regularly suffer from cyberbullying*” (Latvian CA).

Now refuted research by Wakefield strengthened the anti-vaccination movement in Europe. However, despite the paper being disproved and withdrawn, and the author being struck off the medical profession, the paper nevertheless affected certain policy decisions. In Italy in 2000, a judge

“*has imposed the Ministry of Health to indemnify an autistic boy from Agrigento who in 2000 had taken the tetravalent*” (Italian CA).

International discussion on vaccination safety formed part of the background discussion of the Romanian epidemic outbreak of 026: H11 E Coli and its potential link with a vaccination and in the Czech Republic and Croatia where the mandatory immunisation was debated.

The importance of evidence in planning child health policy and care was observed in many national and international initiatives; and debates were supported by locally-published data and statistics. In the Netherlands, reports about the sexual abuse of children were a consequence of an investigation of the Samson Committee and provoked nationwide debate about sexual abuse. An important role was also played by the fact that:

“*in more European countries and the USA there was a surge in reports of sexual abuse in the Roman Catholic Church. The more people reported on their history of abuse, the more people joined in with their own report*” (Dutch CA).

In the UK, the discussion about child sexual exploitation took into account the number of ongoing inquiries such as the Rochdale report, the Rotherham Report and on gangs and gang-related behaviour. In Italy, the illegal dumping became a national and European issue.

#### Cross-nationality

The surge in global and national reports is correlated with the cross-border nature of many of the child health policy and care issues. Issues such as obesity, vaccination, child abuse or care for migrant children are not restricted within the border of any one country but are part of global issues of lifestyle changes, increased awareness of personal health thanks to better communication and increased awareness of children’s rights. Often, such cross-national comparison resulted in exchange of views and ideas and learning from the experiences of other countries.

#### Harmonisation and unification

Cross-national public concern about children increases with the development of international organisations, such as the European Union, which require regions and countries to undertake some form of harmonisation and unification. The need for national adaptation of European regulations supplemented the debates in the Czech Republic about changes in the organisation of child and adolescent health care services. The proposed merge of two medical specialities in consequence of the:

“*call for the need to harmonise medical education with other EU countries. The number of specialisations should, therefore, be reduced from the current 46 to 33 fields and the length of their studies shortened*” (Czech CA).

In order to reduce children’s exposure to passive smoking in Latvia, the measures undertaken were adaptations of European Union legislation [12].

Croatia, as one of the signatories of the UN Convention on the Rights of Persons with Disability in 2007, was obligated to respect its rules. Therefore, when the rights of children with disabilities and their caregivers were changed to their disadvantage by introduction of a new law:

“*this convention was frequently cited in the protests and demands of the non-profit organisation for children with disabilities’ rights*” (Croatian CA).

#### Global processes and movements

The global processes and movements affected many national discussions. One of the most influential was the global economic crisis which influenced the functioning of children’s health care services and policy in most European countries. In particular, Spain, Portugal, Greece, Malta and Ireland struggled with child poverty and homelessness. The global humanitarian crisis and the plight of unaccompanied asylum seekers, discussed previously, was prominently occurred mainly in the UK and Finland; and the situation of migrant families worsened in countries affected by the economic crisis. Globalisation contributed to diagnosis and treatment options that were the subject of national debate. In Iceland, for example, the gradual increase in the use of methylphenidate to treat children with ADHD was controversial.

“*The controversy was about the increase in use of medicalization of normal behaviour of children or a new phenomenon in child development or a problem that has hitherto been unrecognised. The discussion has been fuelled by media reports and information campaigns by patient groups and parent groups*” (Icelandic CA).

Globalisation and global advertising strategies have stimulated debate about worsening mental health among children in Germany. The case referred to the use of digital media, such as smartphone addiction and its impact on children and adolescents.

### Situational aspects

On occasion it was a single incident or situation that prompted national discussion and facilitated policy creation or change. Examples of these phenomena were grouped into: global events, behavioural events, procedural, and institutional events.

#### Global events

Some debates were started or intensified as a result of situation specific events; and then correlated to global events described previously.

#### Behavioural events

Behavioural episodes are correlated with bottom-up initiatives and actions which affect the wider discussions. In Iceland, for example, the debate on child abuse was stimulated by:

“*one case, which involved two boarding schools (Breiðavík and Silungapollur), schools for children/adolescents with problematic backgrounds, but has now been closed since around the 1970s. Another case involved child sexual abuse in a Catholic school and another in a public primary school. In all cases, it is a mixture of physical, psychological and sexual abuse. Neglect has also been on the agenda, many reports, but not all verified*” (Icelandic CA).

Vulnerable children were discussed in the Czech Republic, as a result of the case of “Eva Michálková vs. Norwegian Barnevernet”. A woman’s two sons were taken into protective care after the child reported to a nursery teacher that the father “groped inside his pyjamas” [13]. In consequence, she lost the parental rights to her two sons.

In France, there is a rise in vaccine hesitancy. This was partly a consequence of an inadequate response to the H1N1 (flu) pandemic of 2009 and a lack of compliance with medical procedures which resulted in a lack of trust in vaccination programmes.

“*One of the main errors was to not associate GPs in mass vaccination, although they are known to be key actors in the final decisions regarding vaccination among their patients. But before, controversies about the hepatitis B vaccination deeply damaged the public trust in governmental decisions about vaccinations. France is an exception concerning the vaccine against hepatitis B. It is the only country among those where vaccination is practised, to have had a controversy about the safety of the vaccine, which has lasted from mid-1990*” (French CA).

The nurses’ protest in Poland was the final result of long-lasting unsuccessful negotiations that parents had been undertaking with the government.

“*The demands of protesting parents from the beginning concerned the increase in the amount of care benefits to the level of the minimum wage in Poland, official recognition of the care of disabled children for work (and caregivers as assistants for disabled persons) and an increase in the amount of benefits for people with disabilities: attendance allowance and disability pension. The first protest took place in 2009 and was organised by the parents joined in the frame of the “Together We Can Do More” Forum. In the protest parents from many places in Poland spontaneously took part*” (Polish CA).

The increased prevalence of cancer in Italy, including amongst children was correlated with the presence and burning of toxic waste. This illegal practice was widely believed to be a consequence of organised crime.

#### Procedural and institutional events

The procedural and institutional events were often correlated with the introduction of a new law, which intensified the discussion on a specific issue. For example, the French law on equal marriage (also called the Taubira law) gave new rights for marriage, adoption and inheritance in the name of equality and shared freedoms. Thus became significant in the debate on contraception, in particular, emergency contraception.

The fire in the Romanian hospital occurred during the economic crisis, but what was shocking to the public, was that simple safety procedures were not in place:

“*it occurred one year after the prime minister of that period made public an engagement of rehabilitation of 22 maternity sections in 16 counties of the country. The Giulesti hospital “Panait Sarbu” is evaluated as the highest level possible - hospital level III - because of the technological equipment and highly-qualified medical personnel, and in spite of this excellent evaluation the fire claimed so many victims, as there was no smoke alarm system, and the procedures for evacuation of patients were not known to the personnel; there were deficiencies in the periodic control of the electric system (fire broke out due to an improvised socket that connected the source of electricity to the air conditioner)”* (Romanian CA).

The introduction of new regulations or parliamentary decisions sparked many national debates. In Finland, for example, the debate on sexual education and the reluctance of some religious groups to cover information on sexual and gender minorities was fuelled by the acceptance of the gender-neutral marriage act in 2015″.

The proposal of new vaccination policies in Estonia, linking the disbursement of family benefits with periodical visits to the family physician, was directly linked to the:

“*coalition agreement of 2015 and the article that mentions the clause of the agreement regarding the linking of family benefits with visits to the family physician*” (Estonian CA).

In Lithuania, the proposal of the restricted vaccination plan corresponded to the order on hygiene standards of the Lithuanian Minister of Health which stated that an institution that performs a nursery school and /or pre-school education programme has the right to demand the evidence of the child’s vaccination status. If a child has not been vaccinated against measles, rubella and polio, a child cannot attend. The debate remained in the population for 2 years, despite the Constitutional Court ruling in 2016, that this order is against the Lithuanian Constitution.

In Spain, the action accompanying the debate on child poverty was undertaken by:

“*NGOs and health care and social protection organisations. The call for action addressed increasing unemployment, inequalities and groups at risk/vulnerable individuals like children and minorities/migrant families*” (Spanish CA).

Public outcry against the practice of sedating children in residential homes in Romania has influenced data gathered by the Centre for Legal Resources; which compiled:

“*a long list of Romanian family type placement centres for children with disabilities and found that around half of the children looked after in these centres were medicated, for example, with Rispen (Risperdal, Risperidone) or Stratera for ADHD and other medication. After revealing the situation in Brasov, the National Authority for Child Protection asked for a re-evaluation of the psychiatric examination, and the results confirmed that the 12 children needed the initially-prescribed medication, the assessment and treatment being correct, administration should be continued*” (Romanian CA).

In the UK, Child sexual exploitation was investigated in Northern Ireland, and:

“*linked to Operation Owl, an investigation in missing persons in Northern Ireland. During the course of the investigation, it was uncovered that a higher number of children were going missing more often than others – 13 individual children accounted for 10% of all missing persons, and a further 40 had been reported missing more than 25 times in less than a year and a half. This then resulted in a special investigation into these children*” (UK CA).

In 2013, Operation Owl led to the identification of 22 suspected cases of child sexual exploitation.

## Discussion

The importance of context in the process of children’s health care and policy making is more significant than ever, and this research demonstrates which contextual factors play regulatory function towards child health policy. In our study, we revealed clusters of neutral health policy determinants which we hope that will contribute to the provision of the child health care of high quality, with a strong recognition of contextual factors which might facilitate or impede (if not recognized) the policy performance.

Our research identifies the socio-cultural determinants of children’s health care services and policy, which consist of elements such as societal activation, awareness, communication, trust, freedom, tolerance and religion, history, change, and lifestyle.

The level of societal activation in a country determines the initiatives within child health policy and often is driven by public sensitivity about child related issues. It refers to the extent of social stimulation regarding changes within child health policy. Societal activation can be seen in two dimensions: as public involvement in policy modifications, or as a lack of involvement. We observed it may have two forms such as individual activism and mass activation. The multiple voices of society can result in the introduction of new procedures, action plans and guidelines; influence the level of awareness, intensify scrutiny, increase access and availability of services and prompt the introduction of structural changes or the withdrawal of unfavourable changes. This is possible thanks to the high level of societal awareness and appropriate communication between the policymakers and citizens. Such approach helps to build the relationship based on mutual trust and respect for shared values embedded for example in religion and history. The tolerance together with appreciation of freedom based on aware consideration of profit and loss is a key for introduction of the change within child health policy in order to reinforce pro-health choices (pro-health lifestyle).

The significance of socio-cultural determinants in child health policy, further reasserted by the WHO value-based approach, which recognises the importance of cultural aspects in person-centred health policy [14, 15].

Our classification of internal and external structural determinants brings the reflection on the importance governance for health which is understood as “joint actions of health and non-health sectors, of public and private sectors and of citizens for a common interest” as Kickbush and Gleicher [16] define it. The importance of determinants external to the health care system such as politics, policy and economy strengthen the assumptions of WHO Framework which promotes health in all policies, “an approach on health-related rights and obligations” which “improves accountability of policymakers for health impacts at all levels of policy-making. It includes an emphasis on the consequences of public policies on health systems, determinants of health, and well-being” [17].

Both the socio-cultural and structural determinants of child health policy developed in our research are consistent with the work of Pfadenhauer et al. [4], who described seven domains of context: geographical, epidemiological, socio-cultural, socio-economic, ethical, legal, and political context.

International determinants of child health policy identified in our research reflect the current situation at global level. Global processes and movements are not insignificant in relation to child health policy in Europe. Many countries struggling with the problem of restrict austerity or unexpected influx of migrants had to adapt national health policy solutions to the new reality. Our data shows that many global trends are reflected in globally-published evidence. This may be in the form of unique data, not always available nationally, which exposes a national problem. International comparison and discussions can also help achieve the resolution of a problem. The cross-nationality of children’s health policy issues underlines the importance of the international context. It is an opportunity to facilitate experience exchange and sharing but it also may require a degree of subordination to international protocols which in Europe has provoked discussions on the independence and autonomy of national health systems. This is related to the concept of Europeanization, introduced by Radaelli (2003) who interpreted it as a “series of top-down and bottom-up processes affecting both formal and informal rules as well as procedures, policy paradigms, styles and shared beliefs and norms” [18].

Situational factors correlate with the determinants mentioned previously. They might influence the shifts in child health policy making by affecting socio-cultural, structural and international issues. The situational context takes into account global, national and local events which strongly affect the way that policy is being done at national level. Contextual determinants react to a constantly evolving European reality, and as a result, policy makers need to account for behavioural, procedural, institutional and global influences during the policy making process. Behavioural events reflect the individuals’ behaviours which affect and provoke initiatives at national level, in other words, the movements at microstructure level cause the actions at macro-structural level. In consequence, they can provoke procedural and institutional actions combined with policy changes, and those related to the institution’s movement and decisions.

### Limitations

Research on context is challenging because of the changing nature of contextual factors. In our research we explored these determinants by analysing case studies identified by national experts. However our data does not include all European countries, as we had no responses from six countries. The nature of the research was qualitative; therefore we recognise that the strong public and professional discussions related to children’s health services in European countries, were identified based on the national expert’s choice and are not necessarily representative of the national situation. Our goal was not to measure quantitatively, but to qualitatively investigate the key motivating factors of policy-making and explore how these affect policy action on children’s health services.

### Practical implications

Context in the process of children’s health care and policy making is significant in that contextual determinants have an active influence on children’s health policy.

By identifying and presenting characteristics of the contextual determinants, we provide a guide for child health policy makers, allowing them to recognise the contextual influence on child health policy making.

- The way the policies are initiated should recognize the societal values such as tolerance, religion or history as these can affect the level of acceptance of the children’s health policy.
- While developing new initiatives, the needs of the population including lifestyle choices and challenges need to be taken into account. Political sensibility to community needs is crucial.
- The formulation of new policies should be based on evidence, and take into account cross-national influences from European harmonization and unification, not only in the legal sense but also in terms of shared values.
- Negotiating the character of proposed child health policy changes should take into account the voices of an aware and active society.
- Communication at any stage of policy making should be transparent, this builds trust and provides the reassurance that freedom of speech and freedom of choice; and that the best solution is agreed upon. The media is an important channel of communication for both policy makers and policy recipients.
- Implementing the policy should respect the issues of access to care and provision of care to ensure good functionality of the health system. Additionally, policy implementation should take into account the wider context of health in all policies and prioritizing health issues amidst economic decision-making.
- Evaluating child health policy is essential to build the culture of evidence use as a fundamental factor in policy development in European countries.

## Conclusions

Context of child health policy matters in the formulation and implementation of policies. By recognising the important role of societal movements, we may conclude that the regulatory function of contextual determinants have a broad institutional impact. The multiple voices of society have resulted in the introduction of new procedures, action plans and guidelines; influenced the level of awareness, intensified scrutiny, increased access and availability of services and provoked introduction of structural changes or withdrawing unfavourable changes. Such initiatives were affected, supported and provoked by the variety of contextual factors identified in our research.

## Data Availability

Under Horizon 2020 funding rules the European Commission requires all data to be accessible to the greatest degree possible, but also recognising the potential conflict between Openness and Confidentiality. To that the MOCHA team have added the importance of Comprehension, as source data cannot be interpreted without clear understanding of the method of acquisition. The MOCHA data contains no patient information, but may contain other personal or institutional data such as source of a commentary. The MOCHA project has therefore resolved that source data will be curated on the MOCHA web site, and be accessible via the Principal or other Partners through a curator function, so that data relevant to any enquiry can be supplied, and redaction effected, but also contextualisation given. The datasets used and/or analysed during the current study are available from the corresponding author on reasonable request.

## Abbreviations

ADHD: Attention deficit hyperactivity disorder
CA: Country Agent
EEA: European Economic Area
EU: European Union
GDP: Gross domestic product
GP: General practitioner
HSBC: Health Behaviour in School-Aged Children
MMR: Measles, mumps, and rubella vaccine
MOCHA: Models of Child Health Appraised
NGO: Non-governmental Organization
OECD: Organisation for Economic Co-operation and Development
UASCs: Unaccompanied asylum seekers
WHO: World Health Organization
